# Validation of saliva sampling as an alternative to oro-nasopharyngeal swab for detection of SARS-CoV-2 using unextracted rRT-PCR with the Allplex 2019-nCoV assay

**DOI:** 10.1099/jmm.0.001404

**Published:** 2021-08-09

**Authors:** Marco Andres Bergevin, Wesley Freppel, Guylaine Robert, Georges Ambaraghassi, Dany Aubry, Olivier Haeck, Maude Saint-Jean, Alex Carignan

**Affiliations:** ^1^​ Department of Medical Biology, Hôpital Cité-de-la-Santé, Laval, QC H7M 3L9, Canada; ^2^​ Institut National de la Recherche Scientifique, Centre Armand-Frappier Santé Biotechnologie, Laval, Quebec, Canada; ^3^​ Department of Microbiology, Immunology and Infectious Diseases, Faculty of Medicine, Université de Montréal, Montreal, Quebec, Canada; ^4^​ Optilab Laval-Laurentides-Lanaudière, Quebec, Canada; ^5^​ Department of Microbiology and Infectious Diseases, Faculté de Médecine et des Sciences de la Santé, Université de Sherbrooke, Quebec, Canada

**Keywords:** detection, RT-PCR, saliva, SARS-CoV-2

## Abstract

**Introduction:**

The current severe acute respiratory syndrome-associated coronavirus-2 (SARS-CoV-2) pandemic has stressed the global supply chain for specialized equipment, including flocked swabs.

**Hypothesis:**

Saliva could be a potential alternative specimen source for diagnosis of SARS-CoV-2 infection by reverse-transcriptase PCR (RT-PCR).

**Aim:**

To compare the detection efficiency of SARS-CoV-2 RNA in saliva and oro-nasopharyngeal swab (ONPS) specimens.

**Methodology:**

Patients recruited from hospital provided paired saliva and ONPS specimens. We performed manual or automated RT-PCR with prior proteinase K treatment without RNA extraction using the Seegene Allplex 2019 nCoV assay.

**Results:**

Of the 773 specimen pairs, 165 (21.3 %) had at least one positive sample. Additionally, 138 specimens tested positive by both sampling methods. Fifteen and 12 cases were detected only by nasopharyngeal swab and saliva, respectively. The sensitivity of ONPS (153/165; 92.7 %; 95 % CI: 88.8–96.7) was similar to that of saliva (150/165; 90.9 %; 95 % CI: 86.5–95.3; *P*=0.5). In patients with symptoms for ≤ 10 days, the sensitivity of ONPS (118/126; 93.7 %; 95 % CI: 89.4–97.9) was similar to that of saliva (122/126; 96.8 %; 95 % CI: 93.8–99.9 %; *P*=0.9). However, the sensitivity of ONPS (20/22; 95.2 %; 95 % CI: 86.1–100) was higher than that of saliva (16/22; 71.4 %; 95 % CI: 52.1–90.8) in patients with symptoms for more than 10 days.

**Conclusion:**

Saliva sampling is an acceptable alternative to ONPS for diagnosing SARS-CoV-2 infection in symptomatic individuals displaying symptoms for ≤ 10 days. These results reinforce the need to expand the use of saliva samples, which are self-collected and do not require swabs.

## Introduction

The current severe acute respiratory syndrome-associated coronavirus-2 (SARS-CoV-2) pandemic has challenged healthcare systems worldwide in an unprecedented manner. Rapid spread of this virus, with many individuals being contagious yet asymptomatic, has created the need for mass screening, mainly by reverse-transcriptase PCR (RT-PCR). This has stressed the global supply chain for specialized equipment, including flocked swabs, transport media, RNA extraction reagents, commercial RT-PCR tests and dedicated equipment. These limitations have led researchers to develop alternative PCR protocols and specimens for diagnosis [1, 2].

Currently, oro-nasopharyngeal swabs (ONPS) are the preferred specimens for detecting SARS-CoV-2 infection in the province of Quebec, Canada, and in other jurisdictions. Many other jurisdictions have chosen nasopharyngeal swabs (NPS) as standard specimens for diagnosis. However, both NPS and ONPS present several difficulties. For most individuals, this procedure is uncomfortable; in others, the process is extremely painful, which may decrease the willingness to undergo retesting, particularly in asymptomatic individuals [3]. Furthermore, infection control measures to mitigate nosocomial coronavirus disease-19 (COVID-19) transmission may require healthcare workers (HCWs) to be repetitively screened, compounding the issue of discomfort associated with ONPS. The procedure may stimulate coughing or sneezing, and requires proximity, posing theoretical risks of infection for frontline HCWs.

Salivary sampling can circumvent many of the difficulties associated with oro-nasopharyngeal swabbing. Saliva can be reliably self-collected without posing risks to others, is painless to produce and does not rely on swabs. This specimen source has already been explored for diagnosing upper respiratory tract viral infections, yielding reliable results and decreasing the specimen collection time for HCWs [4, 5]. The current pandemic has sparked interest in salivary testing for the diagnosis of SARS-CoV-2, and previous studies showed the potential of using saliva as a specimen in both symptomatic and asymptomatic patients [6–9].

In this study, we evaluated saliva as an alternative specimen source for detecting SARS-CoV-2 RNA compared to ONPS.

## Methods

### Population and design

We prospectively evaluated saliva as a specimen for detecting SARS-CoV-2 RNA as compared to standard ONPS specimens. This study was conducted in the Laval region of Quebec, Canada, and patients were recruited from 28 April 2020 to 2 November 2020. We studied patients with both a known and unknown SARS-CoV-2 infection status. Patients with an unknown infection status were recruited from dedicated screening centres in Laval and from the emergency department of Cité de la Santé, a 500-bed community hospital with an emergency department receiving over 85 000 visits per year.

Patients with a known infection status were recruited from the emergency department, the COVID-19 designated wards of Cité de la Santé hospital, and from their homes, over the phone and quarantine centres. The patients recruited in the hospital provided paired saliva and ONPS specimens. However, the patients recruited at home gave only a saliva specimen, which was collected within 48 h after collecting a positive ONPS in a dedicated screening centre. For all patients, the symptomatic or asymptomatic status was recorded; for those with symptoms, the number of days since symptom onset at the time of testing was recorded.

The Cité de la Santé hospital’s institutional review board approved this study, and all patients provided informed verbal consent after being handed a patient information sheet explaining the study and how to collect a saliva specimen.

### Specimen collection and processing

ONPS were collected with flocked swabs by first swabbing the throats of the patient and then the nasopharynx with the same swab. The specimens were placed in 1–3 ml of molecular-grade water, refrigerated on-site and sent to the lab in coolers with ice packs. Approximately 5 ml of drooled saliva was collected in sterile plastic containers and sent refrigerated to the laboratory.

Our testing method for ONPS in manual plates was previously described in detail [10]. The specimens were either analysed through manual PCR plating or automated PCR plating with slight variations in the protocol, which have been validated to give similar results in our lab. The laboratory used to analyse the samples was maintained at room temperature around 20–25 °C. After vortexing the collection tube with the flocked swab, the specimen was diluted 1 : 1 with a solution of molecular-grade water containing 200 µg ml^−1^ of proteinase K (PK) solution (ref. 25530049; Thermo Fischer Scientific). For manual plating, specimen dilution was performed in microplates, which were then heated in a thermal cycler for 15 min at 50 °C to permit PK digestion followed by 3 min at 90 °C for PK inactivation and thermal lysis. For automated plating, 300 µl of the specimen was combined with 300 µl of PK solution in a 1.5 ml screw-top conical tube and heated in a dry bath for 20 min at 50 °C and then for 12 min at 95 °C (time and temperature were optimized to mimic activation and inactivation of manual plating). In both protocols, 8 µl of treated specimen (in place of extraction eluate) was added to the PCR wells for direct testing with the Allplex 2019-nCoV Assay (Seegene) [10]. The nCoV assay detects three viral genes: *E (Envelope*), *RdRp (RNA-dependent RNA polymerase*) and *N (Nucleocapsid*). Only *N* is typically detected when the viral load is low [cycle threshold (Ct) ≥ 35]; therefore, we used the *N* Ct values to compare ONPS and saliva samples [10].

The saliva specimens were analysed in a similar manner, except that the dilution with PK solution was 1 : 4 in the first part of the study, which was modified to 1 : 2 in the later stages of the study. A 1 : 1 dilution yielded many invalid results (failure to amplify the internal control) in preliminary evaluations with the saliva specimens.

The specimens yielding invalid results were first retested by the standard method without extraction. If they were invalid a second time, they were retested after extraction with the Seegene Starlet (Seegene) according to the manufacturer’s instructions.

### Statistical analysis

Data were analysed using Stata/MP 15.1 for Mac. Proportions were compared using the chi-squared test. Continuous variables were compared using Wilcoxon’s rank-sum test. We determined the sensitivity of both ONPS and saliva, along with their 95 % confidence intervals (CIs). To compare ONPS and saliva, we used Ct of the *N* gene and ΔCt on paired samples, and statistical significance was assessed by a paired *t*-test.

## Results

A total of 773 specimen pairs were available for analysis. Of these, 681 pairs belonged to the screening group with unknown COVID status, and 63 pairs belonged to the known COVID group. Twenty-two patients with known COVID-positive status submitted saliva samples collected within 48 h of ONPS sampling; they were included only in qualitative analysis and excluded from any analysis comparing Ct values between the specimens.

Of the 773 specimen pairs, 165 (21.3 %) had at least one positive sample. SARS-CoV-2 was detected in 85 women (51.5 %) and 80 men (48.5 %), and the median age was 44 years (interquartile range: 31–58 years). Additionally, 148/165 (89.7 %) patients were symptomatic, and 17/165 (10.3 %) were asymptomatic. For symptomatic patients, the median duration of symptoms was 3 days (interquartile range, 2–7 days), 116/148 (78.4 %) of symptomatic patients had symptoms for 7 days or less, 10 patients had symptoms for 8–10 days, and 22 patients had symptoms for more than 10 days.

A total of 138 specimens tested positive by both sampling methods. Fifteen and 12 cases were detected only from ONPS and saliva specimens, respectively. The sensitivity of ONPS (153/165; 92.7 %; 95 % CI: 88.8–96.7) was similar to that of saliva (150/165; 90.9 %; 95 % CI: 86.5–95.3; *P*=0.5). To further investigate the role of symptom duration on sensitivity, we compared both sampling methods. In patients with symptoms for ≤ 10 days, the sensitivity of ONPS (118/126; 93.7 %; 95 % CI: 89.4–97.9) was similar to that of saliva (122/126; 96.8 %; 95 % CI: 93.8–99.9 %; *P*=0.9). However, the sensitivity of ONPS (20/22; 95.2 %; 95 % CI: 86.1–100) was higher than that of saliva (16/22; 71.4 %; 95 % CI: 52.1–90.8) in patients with symptoms for > 10 days. Asymptomatic patients showed no significant difference between the sensitivity of ONPS (15/17; 88.2 %; 95 % CI: 72.9–100) and saliva (12/17; 70.6 %; 95 % CI: 48.9–92.2; *P*=0.20).

The results in the cohort of patients with an unknown COVID status yielded results similar to those of the overall cohort. For ONPS, 137/148 positive cases were detected with a sensitivity of 92.6 % (95 % CI: 88.3–96.9), and in saliva specimens 137/148 cases were detected with a sensitivity of 92.6 % (95 % CI: 88.3–96.9) (*P*=1.0).

### Comparison of cycle threshold for *N*

**Fig. 1.**
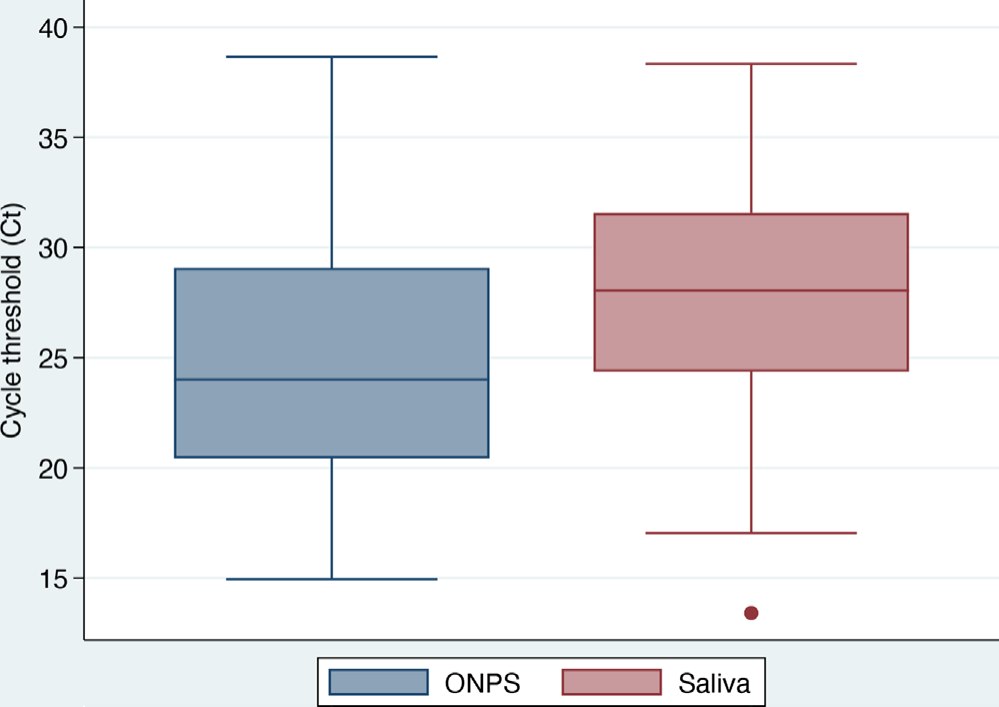
Distribution of mean cycle threshold (Ct) among COVID-positive patients according to sampling method.

A total of 119 patients had both positive concordant pairs and sample collection in the same time frame (Fig. 1). In these patients, the mean Ct for ONPS-positive specimens (25.0; 95 % CI: 24.0–26.0) was lower than that for saliva-positive specimens (28.1; 95 % CI: 27.1–29.0) (Δ3.1; 95 % CI: 2.0–4.1; *P* < 0.001).

There were 27 discordant pairs (Table 1). The average Ct value of *N* for 15 ONPS+/saliva− pairs was 32.7 (95 % CI: 30.3–35.5), and only three patients had Ct values under 30. The average Ct value for 12 ONPS−/saliva+ pairs was 32.6 (95 % CI, 29.7–35.4), and only three patients had Ct values under 30. For discordant symptomatic cases, the median number of days since symptom onset was 7.5, which is higher than the median of 3 days for patients with concordant results (*P*=0.001). In patients with symptoms for more than 10 days, 36 % (8/22) patients had discordant results vs. 9 % (12/126) of patients with symptoms for 10 days or less (*P*=0.003). There was also an overrepresentation of asymptomatic patients in our discordant pairs, representing 25.9 % (7/27) of the discordant pairs vs. 7.3 % (10/138; *P*=0.009) of pairs in patients with concordant results.

**Table 1.** Characteristics of discordant pairs

Patient no.	Gender	Age (years)	Days with symptoms	*N* Ct values	
				ONPS	Saliva
Positive only in ONPS					
1	F	32	2	21.49	
2	M	20	3	39.26	
3	F	43	6	35.15	
4	M	32	7	35.49	
5	F	60	11	32.75	
6	M	61	11	29.92	
7	M	33	16	36.39	
8	M	60	16	32.9	
9	F	70	17	31.54	
10	F	64	21	38.58	
11	M	26	Asx	35.58	
12	F	47	Asx	30.31	
13	F	76	Asx	34.18	
14	F	57	Asx	25.43	
15	F	62	Asx	34.11	
Positive only in saliva					
1	M	56	1		35.76
2	M	31	2		37.28
3	M	53	3		23.92
4	F	37	3		29.73
5	F	39	7		24.38
6	F	82	7		35.2
7	F	90	8		32.99
8	F	26	10		32.2
9	M	38	14		33.17
10	M	84	26		35.51
11	M	20	Asx		33.74
12	F	11	Asx		37.15

Asx, asymptomatic.

As shown in Table 2, we tested the stability of a subsample of 10 salivary specimens at room temperature after various times (median 8 days). The mean Ct for *N* after retesting (32.8; 95 % CI: 30.2–35.3) was similar to the mean Ct after initial testing (32.4; 95 % CI: 29.7–35.1) (Δ0.40; 95 % CI 0.22–1.01; *P*=0.18) and demonstrated that the viral RNA remained stable for as long as 12 days.

**Table 2.** Stability of SARS-CoV-2 RNA in saliva specimens while retesting at various time intervals

Specimen	Initial results (Ct by gene)			Delay (days)	Retest results (Ct by gene)			Delta Ct *N*
	*E*	*RdRp*	*N*		*E*	*RdRp*	*N*	
1	30.88	33.25	32.78	9	31.07	38.23	32.78	0
2	Undetected	Undetected	37.16	6	Undetected	Undetected	38.86	+1.7
3	29.92	31.3	31.55	12	29.85	33.46	31.21	−0.34
4	31.95	33.15	32.69	7	29.7	31.51	31.21	−1.48
5	27.89	29.52	29.44	12	26.9	30.66	28.99	−0.45
6	23.93	26.02	26.13	12	24.52	28.79	25.71	−0.42
7	33.13	35.7	36.16	7	34.08	Undetected	35.44	−0.72
8	31.72	34.36	34.32	7	30.46	33.07	33.14	−1.18
9	Undetected	Undetected	37.09	8	34.57	Undetected	36.23	−0.86
10	29.25	30.52	30.43	8	28.04	32.5	30.21	−0.22

## Discussion

The overall sensitivity of saliva was similar to that of ONPS in a cohort mainly composed of symptomatic patients presenting with symptoms for 10 days or less. These results are reassuring because saliva can be self-collected; thus, fewer qualified HCWs will be required at screening centres. Moreover, saliva collection can be delocalized out of testing centres and can be performed in schools or workplaces where index cases have appeared. This much less invasive and more comfortable sampling method will theoretically promote testing of larger numbers of people. Reduced inconvenience and self-collection indicate the practicality of this method in implementing repeat sampling of cases of outbreaks occurring in healthcare facilities and for routine screening. Models have estimated that the frequency of sampling and turnaround time are the most important factors in SARS-CoV-2 surveillance [11]. We could not improve the turnaround time of saliva sampling, but this method can lead to more frequent sampling becoming much more acceptable to both HCWs and patients. Our results also showed that saliva specimens were stable at room temperature for at least 7 days, as also shown by Wyllie *et al*. [2].

We found that some patients tested positive only in the ONPS specimen, whereas others tested positive only in the saliva specimen, which may be because viral kinetics can differ in various body sites [12]. In agreement with our colleagues from British Columbia [13], in symptomatic individuals with a first negative test-result and held in isolation or in quarantine pending a second test because of high clinical or epidemiological suspicion, both saliva and ONPS sampling may be warranted to increase the overall sensitivity of the test.

Babady *et al*. reported a sensitivity of 96.7 % for saliva samples compared to that of ONPS in a cohort of symptomatic and asymptomatic HCWs [14]. McCormick-Baw *et al*. also demonstrated a 96 % positive agreement and 99 % negative agreement of saliva with respect to NPS, with saliva detecting 48/51 positive pairs and NPS detecting 50/51 positive pairs in a study of 156 paired samples [15]. However, a lower sensitivity (82.9 %) of saliva samples was recently reported in a study conducted in Marseille, France. In contrast to our study, this cohort included mainly asymptomatic patients (60.4 %) and patients with follow-up testing. There were few asymptomatic patients in our study. When low viral loads are encountered in these patients, it was not possible to determine if they have ascending viral loads from a recent infection or if they are in the late stage of infection and are probably non-infectious. Screening of asymptomatic patients is focused on identifying potentially contagious individuals; additionally, as saliva performs as well as ONPS in the first 10 days of symptoms, which covers the period of infectiousness for the general population [16–18], our results are applicable for the screening of asymptomatic patients. Furthermore, in a large cohort (*n*=1924) of asymptomatic individuals screened with NPS and saliva, either for contact tracing or systematically for international travel quarantine in Japan, the sensitivity of saliva and NPS was 92 and 86 %, respectively [8].

Another Canadian study demonstrated the feasibility of salivary detection of SARS-CoV-2 in the setting of a COVID-19 testing centre [19] and found a lower estimated rate of detection relative to swab testing; however, they used a different protocol, where (i) a preservative/viricidal fluid mixture was automatically released into the sealed saliva sample and (ii) the PCR assay was different. In our study, all samples were analysed using unextracted rRT-PCR after dilution, PK treatment and thermal lysis [10]. This approach saves time and costs as compared to the extracted RT-PCR. According to our observations and published data, infectious individuals (presumably with viable virus) present viral loads higher than the limit of detection of the test using saliva samples in our study; thus, it appears that we did not overlook contagious individuals [13, 20–24].

### Limitations

The main limitation of our study was the small number of asymptomatic patients, which hindered our ability to draw some conclusions in this subgroup of patients. Moreover, our results may not be generalizable to all populations, as very young patients aged < 3–4 years as well as patients with neurocognitive disorders may experience difficulty in self-collection. In addition, those afflicted by xerostomia may have difficulty producing a saliva specimen.

## Conclusion

In this prospective study, we have demonstrated that a saliva sample is an acceptable alternative to ONPS samples for diagnosing SARS-CoV-2 infection in symptomatic individuals displaying symptoms for 10 days or less. These results reinforce the need to expand the use of saliva sampling, which can be performed by the patient, involves reduced inconvenience, reduces risks to HCWs and reduces human resources required for testing. Saliva tests may create a paradigm shift in nosocomial outbreak screening, as most patients and HCWs will willingly submit to repeated, even daily testing, permitting rapid exclusion of infected workers and faster implementation of isolation measures for affected patients in ward outbreaks. Finally, saliva testing may also facilitate future scientific studies where repeated home sampling is required by alleviating logistical constraints.
